# Comparative evaluation of hypoxia preconditioned serum (HPS) and platelet-rich plasma (PRP) on preadipocyte survival and adipogenic differentiation

**DOI:** 10.3389/fbioe.2025.1683899

**Published:** 2025-10-20

**Authors:** Jun Jiang, Michael Martin, Lynn Röper, Samuel Knoedler, Vincent Steinbacher, Sarah Alageel, Marc Hanschen, Haydar Kükrek, Ulf Dornseifer, Arndt F. Schilling, Hans-Günther Machens, Philipp Moog

**Affiliations:** ^1^ Experimental Plastic Surgery, Clinic for Plastic, Reconstructive and Hand Surgery, Klinikum Rechts der Isar, Technical University of Munich, Munich, Germany; ^2^ Institute of Molecular Immunology and Experimental Oncology, Klinikum Rechts der Isar, Technical University of Munich, Munich, Germany; ^3^ Cellular Therapy and Immunobiology, Research and Innovation, King Faisal Specialist Hospital & Research Center, Al Mathar Ash Shamali, Riyadh, Saudi Arabia; ^4^ Department of Trauma Surgery, Klinikum Rechts der Isar, Technical University of Munich, Munich, Germany; ^5^ Department of Plastic, Reconstructive and Aesthetic Surgery, Isar Klinikum, Munich, Germany; ^6^ Department of Trauma Surgery, Orthopedics and Plastic Surgery, University Medical Center Göttingen, Göttingen, Germany

**Keywords:** lipofilling, fat graft survival, hypoxia preconditioned serum, HPS, PRP, preadipocyte, regenerative medicine

## Abstract

**Introduction:**

Lipofilling is a widely used technique in plastic and reconstructive surgery, but its long-term success is often limited by unpredictable fat graft resorption. Optimizing the adipogenic environment through bioactive factors may enhance graft survival and volume retention. This study investigates the adipogenic potential of Hypoxia Preconditioned Serum (HPS) and Platelet-rich Plasma (PRP), in comparison to normal serum (NS).

**Methods:**

Cytokine profiles of HPS, PRP, and NS from 10 donors were analyzed. Human preadipocytes (n = 3) were cultured with low (10%) and high (40%) concentrations of these secretomes. Proliferation, cytotoxicity (LDH assay), lipid droplet formation (Oil Red O staining), and gene expression (qPCR) of adipogenic markers (PPARgamma, C/EBPalpha, FABP4, Adiponectin, LPL) were assessed after 2 and 4 days.

**Results:**

HPS contained significantly higher levels of Adiponectin, IGF-1, bFGF, VEGF-A, and PDGF-BB compared with PRP and NS, while Leptin was lower in HPS and PRP than in NS. All conditions increased proliferation on day 4, with the highest cell counts in NS-40%. No treatment-related cytotoxicity was observed. HPS-40% induced the strongest adipogenic differentiation, evidenced by increased lipid droplet formation and upregulation of all measured adipogenic genes by day 4.

**Conclusion:**

These findings suggest that HPS enhance the proliferation, survival, and differentiation of preadipocytes. Validation in *in vivo* models and clinical studies will be necessary to confirm its potential efficacy in enhancing graft survival and volume retention.

## 1 Introduction

Autologous fat grafting, or lipofilling, is a widely used technique in reconstructive and aesthetic surgery for volume augmentation, soft tissue regeneration, and rejuvenation (Abu-Ghname et al., 2019). However, despite its popularity, a key limitation of this technique lies in the unpredictable resorption of the transplanted fat, with volume loss ranging from 20% to 90% (Shauly et al., 2022; Debuc et al., 2023; Khouri et al., 2014; Doornaert et al., 2019). The long-term survival of grafted adipose tissue is influenced by multiple variables, including the methods used for fat harvesting and processing, the degree of vascularisation at the recipient site, and the ability of cells within the graft to survive under ischemic stress (Strong et al., 2015). Among the various cellular components of adipose tissue, preadipocytes are critical contributors to graft retention and long-term maintenance. Unlike mature adipocytes, which are highly susceptible to ischemia-induced apoptosis and necrosis following transplantation, preadipocytes exhibit greater resistance to metabolic and mechanical stress (Bellini et al., 2017). This is attributable to the fact that preadipocytes are committed progenitor cells within the adipose tissue, developed from adipose tissue-derived stem cells (ASCs), that have the ability to proliferate and differentiate into mature adipocytes which allows for the replacement of lost cells, contributing to graft stability over time (Raposio et al., 2017; Florido et al., 2011). In addition, preadipocytes demonstrate greater resilience to hypoxia and trauma, likely due to their ability to survive without nutrients and their lower oxygen consumption rate compared to mature adipocytes (Raposio et al., 2017; von Heimburg et al., 2004). In addition, preadipocytes secrete a variety of trophic and angiogenic factors such as vascular endothelial growth factor (VEGF), fibroblast growth factor-2 (FGF2), and hepatocyte growth factor (HGF), which play vital roles in promoting neovascularisation and mitigating hypoxia-induced apoptosis, both of which are critical for graft integration and perseverance (Leff and Granneman, 2010; Tchkonia et al., 2007; Kakudo et al., 2015). In addition, numerous studies have demonstrated that better volume retention and survival rates are achieved in fat grafts with higher proportions of preadipocytes compared to those that are predominantly composed of mature adipocytes (Zhang et al., 2018; Kølle et al., 2013). Therefore, given their essential role in adipose tissue regeneration, strategies aimed at supporting preadipocyte viability, proliferation, and differentiation are crucial for improving the clinical outcomes of lipofilling.

A current promising approach is the supplementation of fat grafts with bioactive secretomes derived from peripheral blood. In particular, Hypoxia-Preconditioned Serum (HPS) and Platelet-rich Plasma (PRP) have garnered attention for their regenerative potential due to their rich content of cytokines and growth factors that influence tissue repair and cellular behavior. Of the two, Platelet-rich Plasma (PRP) has been the most extensively studied. It is produced by concentrating autologous platelets from peripheral blood and is known to release a robust cocktail of growth factors, including vascular endothelial growth factor (VEGF), platelet-derived growth factor (PDGF), basic fibroblast growth factor (bFGF), and insulin-like growth factor 1 (IGF-1) upon activation (Pavlovic et al., 2016). These molecules are well recognized for their ability to promote angiogenesis, cellular proliferation, and adipogenic signalling (Lubkowska et al., 2012; Sung et al., 2013; Gao et al., 2018). However, the efficacy of PRP in the context of fat grafting has yielded inconsistent results: While some studies report that PRP enhances ASC proliferation, others indicate that it may inhibit adipogenic differentiation (Chignon-Sicard et al., 2017; Liao et al., 2015), which could undermine graft survival, as long-term retention depends on the survival and differentiation of adipocytes.

HPS, by contrast, represents a novel serum formulation generated by subjecting peripheral blood cells (PBCs) to hypoxic conditions prior to serum extraction. This hypoxic preconditioning activates a survival-related stress response in blood cells, stimulating the release of hypoxia-induced growth factors and cytokines (Hadjipanayi and Schilling, 2013; Hadjipanayi and Schilling, 2014; Jiang et al., 2023; Moog et al., 2020). By mimicking the physiological response of cells under low-oxygen conditions, HPS aims to amplify the regenerative signals required for tissue repair and angiogenesis (Ektoras et al., 2023; Jiang et al., 2022a; Jiang et al., 2022b; Jiang et al., 2023a; Jiang et al., 2023b; Jiang et al., 2024a; Jiang et al., 2024b). Unlike PRP which relies on the degranulation of concentrated platelets to deliver a sharp, early burst of growth factors associated with the hemostatic phase of wound healing, HPS contains a broader range of soluble mediators secreted by hypoxia-activated leukocytes and other PBCs (Jiang et al., 2023; Moog et al., 2020).

Graft sites with an augmented blood supply are associated with improved survival outcomes, since an adequate blood supply is paramount for nutrient delivery, oxygenation, and integration of the transplanted tissue (Xining and Sai, 2024). Recent studies performed by our group have highlighted the pro-angiogenic and lymphangiogenic potential of HPS, demonstrating its capacity to stimulate microvascular network formation in both *in vitro* and *in vivo* settings (Jiang et al., 2023; Jiang et al., 2022b; Jiang et al., 2024a; Moog et al., 2023). These findings emphasize HPS’s therapeutic potential in supporting graft viability through improved vascular support. In addition, other regenerative effects on various cells have been demonstrated, including the proliferation and migration of fibroblasts (Hadjipanayi et al., 2019), and the differentiation of osteoblasts (Jiang et al., 2022a) and chondrocytes (Jiang et al., 2023b). In addition to its capacity to enhance murine, porcine, and human wound regeneration (Ektoras et al., 2023; Jiang et al., 2022a; Jiang et al., 2024a; Hadjipanayi et al., 2019), HPS has been shown to promote bone healing in a three-dimensional chick bone defect model *ex vivo* (Jiang et al., 2024b). Collectively, these findings underscore the broad regenerative potential of HPS and support its application as a versatile biological adjunct that can enhance tissue repair and graft integration across multiple contexts.

Given the multifaceted regenerative properties of HPS and PRP, comparative studies are needed to determine which secretome more effectively supports preadipocyte function in the context of fat grafting. By quantifying the levels of adipogenic growth factors in each secretome, assessing the proliferation and viability of human preadipocytes in response to its supplementation, and evaluating their adipogenic differentiation potential of preadipocytes, we seek to identify the most effective serum for promoting preadipocyte survival and differentiation. The findings could have significant clinical implications, offering insights into enhancing adipose tissue grafting outcomes and also contributing to the broader field of regenerative medicine.

## 2 Methods

### 2.1 Ethical approval

This study was conducted as per the Declaration of Helsinki and the approval of the ethics committee of the Technical University of Munich, Germany (File Nr.: 2023-410-S-NP; date of approval: 06 September 2023). Informed consent was obtained from all blood donors involved.

### 2.2 Production of hypoxia preconditioned serum (HPS)

**FIGURE 1:**
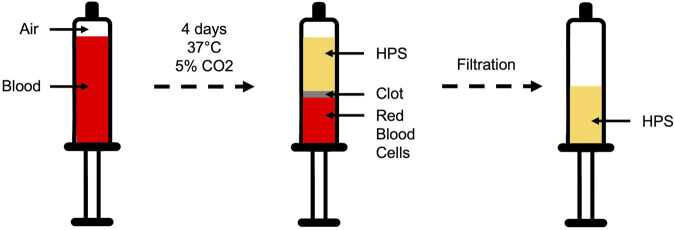
Preparation of Hypoxia Preconditioned Serum (HPS). HPS was generated using a hypoxia-adjusted *in vitro* preconditioning method. Peripheral venous blood was collected, and 5 mL of air was filtered through a 0.2 µm filter into the syringe, which was then placed upright in a temperature-controlled incubator (37 °C, 5% CO_2_). During coagulation and incubation, passive sedimentation separated the sample into three layers: serum at the top, a fibrin clot with peripheral blood cells in the middle, and red blood cells (RBCs) at the bottom. Local pericellular hypoxia (∼1% O_2_) developed within the closed syringe as a result of cellular oxygen consumption. Over 4 days, this environment stimulated production and secretion of cell-derived protein factors into the serum. At the end of incubation, the growth factor–rich HPS was collected and sterile-filtered to remove residual cellular debris.

HPS was produced according to the protocol previously established by our group (Jiang et al., 2024b; Moog et al., 2023). The study included ten healthy human donors—five females and five males—ranging in age from 21 to 34 years. Individuals were excluded if they met any of the following criteria: smoking, pregnancy, presence of systemic inflammatory diseases, or use of any oral medications within 6 weeks prior to blood donation. Briefly, 20 mL of peripheral venous blood was collected into a 30 mL syringe (Omnifix^®^, B Braun AG, Melsungen, Germany), and then 5 mL of air was drawn through a 0.2 µm filter (Sterifix^®^, B Braun AG, Melsungen, Germany). The syringe was subsequently sealed, creating a pericellular hypoxia (∼1% O2) through PBCs’ oxygen consumption during a 4-day incubation period at 37 °C and 5% CO_2_ (Figure 1). Post-incubation, three distinct layers were formed, with the top ‘clear’ layer representing the HPS, which was filtered (Sterifix^®^, B Braun AG, Melsungen, Germany) into a new syringe for further pooled or individual aliquots at −80 °C until experimental testing (for a maximum of 3 months).

### 2.3 Production of normal serum (NS)

Normal serum was obtained from the same individuals who donated blood for HPS preparation: Peripheral venous blood was drawn under sterile conditions and collected into separate 30 mL polypropylene syringes (Omnifix^®^, B Braun AG, Melsungen, Germany). For the preparation of normal serum, the syringes were placed upright for 4 h at room temperature (22 °C) to achieve simple sedimentation. Afterwards, the serum supernatant was filtered (Sterifix^®^, B Braun AG, Melsungen, Germany) into a new syringe using the same procedure as HPS. The normal serum was stored both separately and pooled at −80 °C until experimental testing.

### 2.4 Production of platelet-rich plasma (PRP)

Platelet-rich Plasma (PRP) was prepared using a standardized double-centrifugation protocol (Nagata et al., 2010), with the same donors as those used for the HPS group. 6 mL of peripheral venous blood was collected into 6 mL blood collection tubes (366,575, BD Vacutainer, Becton, Dickinson and Company, Franklin Lakes, NJ, United States), prefilled with trisodium citrate, and centrifuged at 1,300× g for 20 min. This resulted in blood separation into three layers: platelet-poor plasma (top layer), a buffy coat (middle layer containing platelets and white blood cells), and erythrocytes (bottom layer). The upper two layers, which account for 60% of the total blood volume, were pipetted into a new Falcon tube. To minimize any loss of platelets, a few erythrocytes beneath the buffy coat layer were permitted to be collected. A secondary centrifugation of 1800× g ensued for 15 min to separate the bottom PRP (approx. 0.5 mL) from the upper serum component. The serum component was then removed, and the PRP was activated by adding 0.5 mL of 1 I.U./mL Thrombin and 8.88 μg/mL CaCl2 (Tisseel, Baxter, Illinois, United States), which were dissolved in DMEM and FCS 3%. After incubating the mixture for 30 min at 37 °C, a third centrifugation was performed at 2,500× g for 20 min to obtain an activated PRP supernatant, equivalent to the releasate of the PRP-secretome dissolved in DMEM and FCS 3%. PRP was then collected by a sterile syringe and filtered (Sterifix^®^, B Braun AG, Melsungen, Germany) into pooled or individual aliquots, which were stored at −80 °C until experimental testing (for a maximum of 3 months).

To validate the preparation method, platelet counts were compared between PRP and full blood samples from the 10 donors using a C-Chip hemocytometer (DHC-N01, NanoEnTek, Korea). PRP was diluted 1:400 and whole blood 1:200 with PBS; 10 µL of each sample was loaded into hemocytometer chambers, and platelets were manually counted under an inverted phase-contrast microscope (Axio Vert.A1, Carl Zeiss, Jena, Germany). Platelet concentrations were calculated using the manufacturer’s equation. The PRP samples reached a mean platelet concentration of 1,325.13 ± 120.22 × 10^9^/L (vs 264.34 ± 29.47 × 10^9^/L in full blood).

### 2.5 Quantification of adipogenic growth factors

To quantify the secretome growth factors, ELISAs (enzyme-linked immunosorbent assay) were performed using DuoSet ELISA kits (Adiponectin: DY1065, IGF-1: DY291, Leptin: DY398, VEGF-A: 293B, bFGF: DY233, and PDGF-BB: DY220, DuoSet, Bio-Techne Ltd., Minneapolis, MN, United States), following the manufacturer’s protocol. The optical density was measured using a Mithras plate reader (Berthold Technologies GmbH & Co. KG, Bad Wildbad, Germany).

### 2.6 Cell culture

Commercial human subcutaneous white preadipocytes (PromoCell GmbH, Heidelberg, Germany) were used to model committed adipogenic progenitors present in adipose tissue grafts. The cells were cultured in T175 flasks using PromoCell’s preadipocyte growth medium (C-27410, PromoCell GmbH, Heidelberg, Germany) according to the manufacturer’s instructions and were maintained in a humidified incubator at 37 °C with 5% CO_2_ with the medium replaced every 2–3 days. Cells were passaged as needed to obtain the required cell numbers for experimental use. Three donors were utilized for the study: (1) Male, 22 years old, Caucasian, (2) Female, 40 years old, Caucasian, (3) Female, 20 years old, Caucasian. For each experiment, 30,000 cells were seeded in a total volume of 1 mL preadipocyte growth medium in 24-well plates. The plates were incubated at 37 °C with 5% CO_2_ for 24 h to allow cell attachment. Test media included Hypoxia Preconditioned Serum (HPS), Platelet-Rich Plasma (PRP), and normal serum (NS), each at two concentrations: low (10%) and high (40%). The media were prepared by diluting HPS, PRP, or NS in DMEM containing 3% FCS, with the addition of heparin to prevent PRP clotting. The final concentration of heparin was 75 IU in 150 mL DMEM 3% FCS. After aspirating the preadipocyte growth medium, the test media were applied as follows: HPS/PRP/NS at 10% and −40%, negative control (DMEM with 3% FCS), and positive control (preadipocyte growth medium, containing 5% FCS).

### 2.7 Assays

The effects of the test media were evaluated at two time points: day 2 and day 4. All conditions were tested in triplicate.

#### 2.7.1 Cell counting

Cells were washed with PBS, trypsinized with Trysin/EDTA (Trypsin/EDTA Solution 0.25%/0.02% in PBS, Biochrom GmbH, Berlin, Germany), resuspended in 300 μL of DMEM/10%FCS and counted by an automated CASY cell counter (Roche, Mannheim, Germany). The CASY counter technology combines particle identification using resistance measurement with a pulse area analysis based on a digital pulse processing technology. Results of cell counts are given as cells per mL.

#### 2.7.2 LDH assay

Cytotoxicity was determined by measuring lactate dehydrogenase (LDH) release using the LDH Cytotoxicity Assay Kit (Hoffmann-La Roche, Basel, Switzerland). Cell culture supernatant (100 µL) was collected from each well and transferred to a 96-well plate. Corresponding blanks were also included for each condition. A fresh LDH reaction mixture was prepared for 48 wells (calculated for 53 wells to ensure sufficient volume) by combining 2.5 µL of catalyst and 112.5 of µL dye solution per well. After adding 100 µL of the reaction mix to each well, the plate was incubated in the dark at room temperature for 30 min. Absorbance was measured at 490 nm with a reference wavelength of 600 nm using a Mithras microplate reader (Berthold Technologies GmbH & Co. KG, Bad Wildbad, Germany). Absorbance values from blanks were subtracted from the test wells to account for background absorbance.

#### 2.7.3 Oil Red O staining and quantification

Lipid accumulation, as an indicator of adipogenesis, was assessed using Oil Red O staining. An Oil Red O stock solution was prepared by dissolving 150 mg Oil Red O (Sigma Aldrich, St. Louis, United States of America) in 50 mL isopropanol and stirring overnight using a magnetic stirrer. The stock solution was filtered through a 0.2 µm filter and stored at 4 °C. The working solution was prepared fresh each day by mixing 3 parts of Oil Red O stock solution with 2 parts of ultrapure water, vortexing, and then left to sit for 20 min. The solution was filtered through a Whatman filter (Sigma Aldrich, St. Louis, United States of America) and stored in a light-protected container. The culture medium was aspirated, and the wells were washed with PBS. Cells were fixed with 500 µL of 3.7% formaldehyde for 10 min at room temperature, followed by two washes in PBS. Subsequently, 600 µL of Oil Red O working solution was added to each well, and plates were incubated for 15 min at room temperature. The wells were then extensively washed with ultrapure water to remove excess stain. Microscopic images were captured. After imaging, the plates were left to dry overnight. For quantification, 800 µL of 100% isopropanol was added to each well the following day, and plates were incubated for 10–20 min on a shaker. After incubation, 100 µL of the isopropanol solution was transferred to a 96-well plate, and the optical density was measured at 500 nm using a Mithras plate reader (Berthold Technologies GmbH & Co. KG, Bad Wildbad, Germany).

#### 2.7.4 Analysis of gene expression

Total RNA was extracted on day 2 and day 4 using the RNeasy Mini Kit (QIAGEN GmbH, Hilden, Germany) following the manufacturer’s instructions. The purity and concentration of the RNA were checked using a NanoDrop spectrophotometer (Implen GmbH, Munich, Germany). Reverse transcription of RNA samples was carried out using the SensiFast cDNA Synthesis Kit (Meridian Bioscience, Cincinnati, OH, United States) in accordance with the manufacturer’s protocol, utilizing 500 ng of total RNA. The resulting cDNA was used in triplicate RT-PCR reactions. Primers spanning exon–exon junctions were employed to ensure specific amplification of cDNA. Quantitative PCR (qPCR) was conducted with No ROX SYBR MasterMix blue dTTP (Eurogentec, Liège, Belgium). In brief, the reaction mixture—comprising PCR master mix and gene-specific primers at a final concentration of 100 nM—was prepared in a 384-well plate (Fisher Scientific, Waltham, MA, United States). The plate was sealed, vortexed, and centrifuged at 2000×g for 5 min at 4 °C. Subsequently, qPCR was performed on a Roche LightCycler 480 II system (Roche, Basel, Switzerland) using a three-step cycling protocol: carryover prevention at 50 °C for 2 min, initial denaturation and enzyme activation at 95 °C for 5 min, followed by 40 cycles of denaturation at 95 °C for 10 s, annealing at 60 °C for 20 s, and extension at 72 °C for 30 s. Product specificity was confirmed by melting curve analysis. All data were normalized to GAPDH and analysed using the ddCT method. All primers are listed in Table 1.

**TABLE 1:** Primer sequences.

Target gene	Primer forward	Primer reverse
PPARgamma	AGC​CTG​CGA​AAG​CCT​TTT​GGT​G	GGC​TTC​ACA​TTC​AGC​AAA​CCT​GG
C/EBPalpha	AGG​AGG​ATG​AAG​CCA​AGC​AGC​T	AGT​GCG​CGA​TCT​GGA​ACT​GCA​G
FABP4	ACG​AGA​GGA​TGA​TAA​ACT​GGT​GG	GCG​AAC​TTC​AGT​CCA​GGT​CAA​C
Adiponectin	CAG​GCC​GTG​ATG​GCA​GAG​ATG	GGT​TTC​ACC​GAT​GTC​TCC​CTT​AG
Lipoprotein lipase (LPL)	CTG​CTG​GCA​TTG​CAG​GAA​GTC​T	CAT​CAG​GAG​AAA​GAC​GAC​TCG​G
GAPDH	GTC​TCC​TCT​GAC​TTC​AAC​AGC​G	ACC​ACC​CTG​TTG​CTG​TAG​CCA​A

### 2.8 Statistical analysis

Data sets were analyzed using repeated measures of one-way analysis of variance (ANOVA), followed by subsequent comparisons using Tukey’s *post hoc* analysis. If two independent variables were present, two-way ANOVA with subsequent comparisons using Tukey’s *post hoc* analysis was performed. All values are expressed as means ± standard error of the mean (SEM). A value of p < 0.05 was considered statistically significant (*p < 0.05, **p < 0.01, ***p < 0.001, and ****p < 0.0001).

## 3 Results

### 3.1 Quantitative analysis of adipogenic growth factors in different human blood-derived secretomes

**FIGURE 2:**
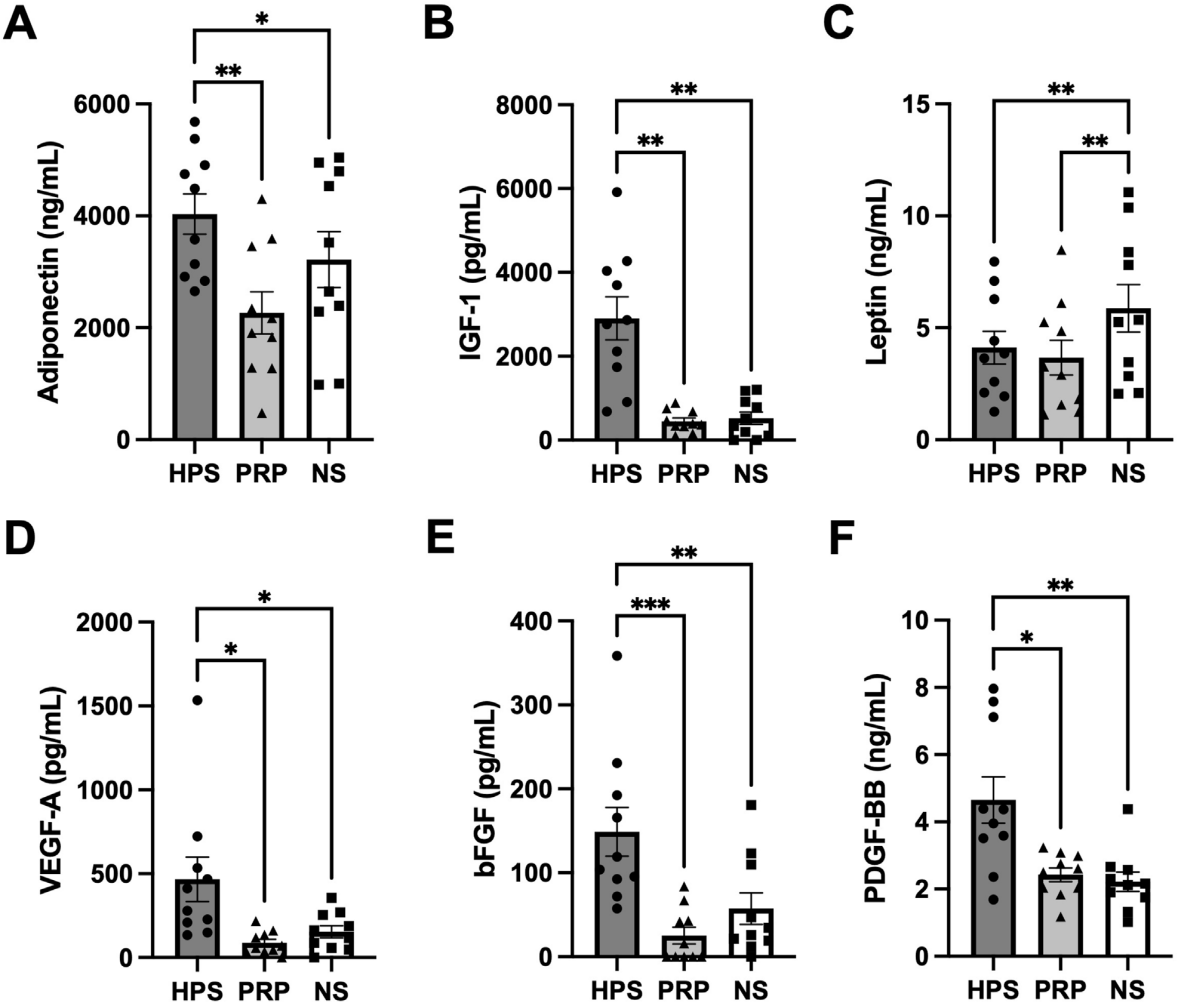
Quantitative analysis of adipogenic growth factors in Hypoxia Preconditioned Serum (HPS), Platelet-rich Plasma (PRP), and normal Serum (NS). **(A–F)** Protein quantification of the **(A)** Adiponectin, **(B)** IGF-1, **(C)** Leptin, **(D)** VEGF-A, **(E)** bFGF, and **(F)** PDGF-BB. One-way repeated-measures ANOVA with Tukey’s multiple comparison test. Data points are means ± SEM, blood donors: n = 10. *p < 0.05, **p < 0.01, ***p < 0.001.

We quantitatively analyzed the levels of adipogenic growth factors in three blood-derived preparations (HPS = Hypoxia Preconditioned Serum, PRP = Platelet-rich Plasma, NS = normal serum). Here, we found that the levels of Adiponectin, IGF-1, VEGF-A, bFGF, and PDGF-BB were significantly increased in HPS compared to NS and PRP (Figure 2). Only leptin was found to be lower in HPS and PRP compared to the NS group.

### 3.2 The effect of different human blood-derived secretomes on the proliferation and viability of preadipocytes

**FIGURE 3:**
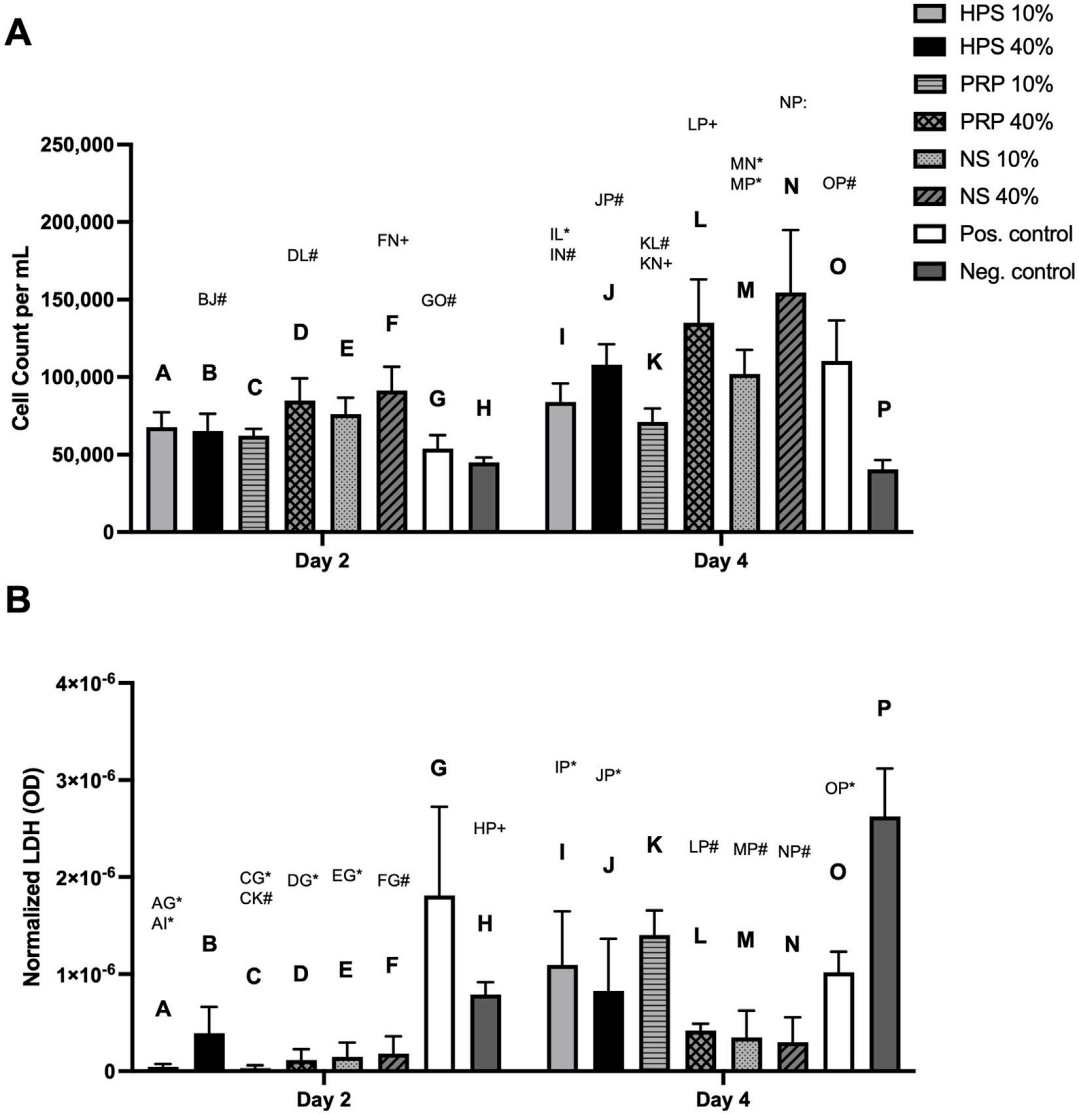
The effect of HPS, PRP, and NS on the viability and proliferation of human preadipocytes. Preadipocytes were stimulated by HPS/PRP/NS-10%, −40%, compared to positive control (growth medium) and negative control (basal medium). **(A)** Cell count after 2 and 4 days of stimulation. **(B)** Lactate dehydrogenase (LDH) assay: optical density (OD) normalized per cell. Two-way repeated-measures ANOVA with Tukey’s multiple comparisons test. Data points are means ± SEM, Preadipocyte donors: n = 3. Capital letter pairs over plots indicate statistical comparison of corresponding data points. For all pair comparisons, * = p < 0.05, # = p < 0.01, + = p < 0.001,: = p < 0.0001.

Next, we investigated the adipogenic effect of the above-mentioned blood-derived secretome on human preadipocytes. We decided to use low (10%) and high (40%) dose concentrations for the following experiments. Here, we found a higher proliferation of all high-dose blood-derived conditions on day 4 compared to day 2 (HPS-40%: 65,308 vs 108,017, p < 0.01; PRP-40%: 84,905 vs. 135,116, p < 0.01; NS-40%: 91,267 vs 154,539, p < 0.001) (Figure 3A). On day 4, the highest cell count was achieved with NS-40% stimulated preadipocytes. Higher doses of PRP- and NS-stimulated preadipocytes proliferated more than the lower dose on day 4 (PRP-40%: 135,116 vs. PRP-10%: 71,078, p = 0.008; NS-40%: 154,539 vs. NS-10%: 102,059, p = 0.04). The HPS-40% condition showed a relatively higher cell count compared to the HPS-10% condition on day 4, but this difference was not statistically significant (p = 0.7). In the LDH cytotoxicity assay, very low values (normalized to cell count) were detected in all conditions on day 2 and day 4 (Figure 3B). There was a relative increase in LDH on day 4 in all the blood-derived conditions; however, they were significantly lower than the negative control (basal media).

### 3.3 The effect of different human blood-derived secretomes on the differentiation of preadipocytes

**FIGURE 4:**
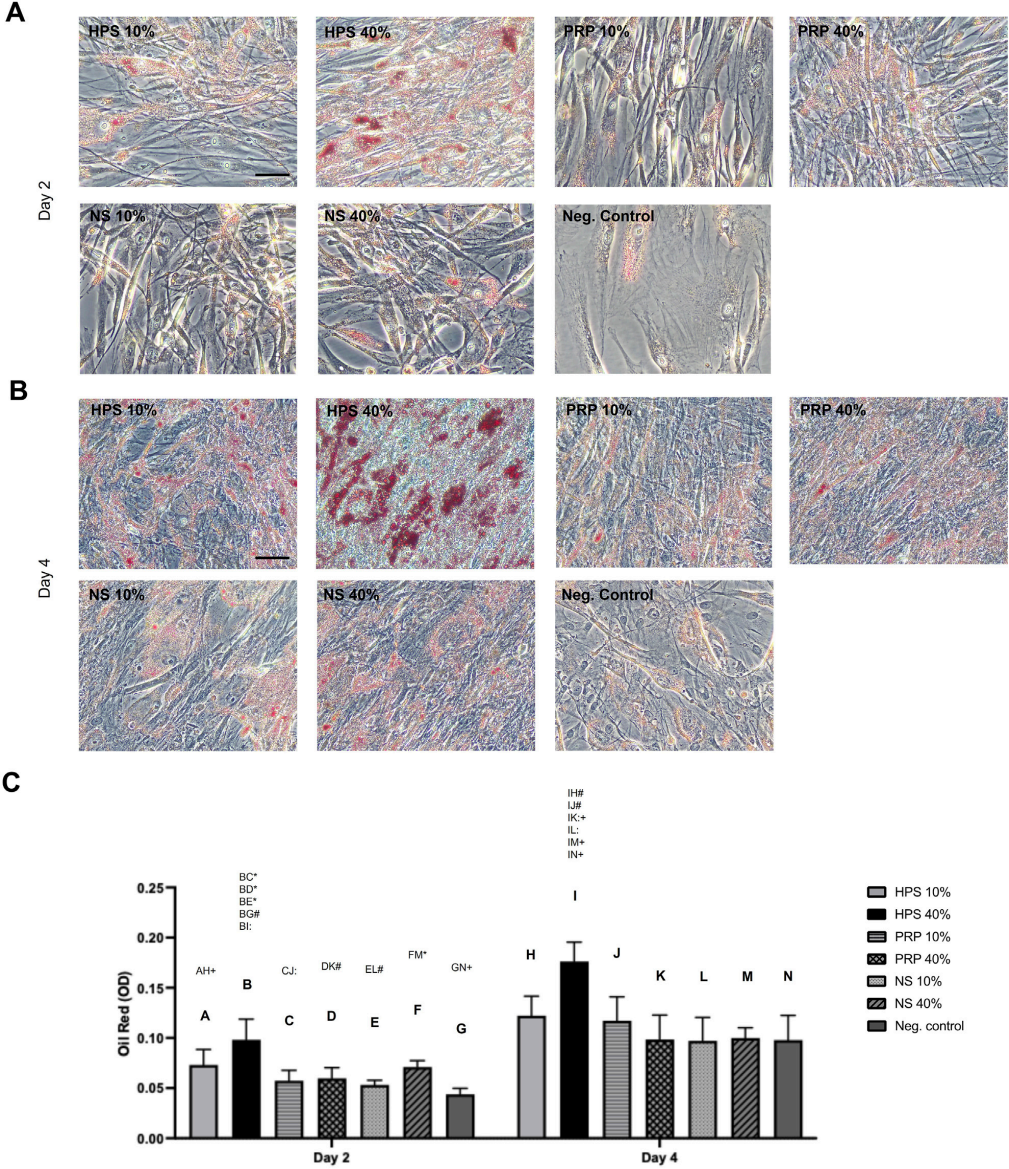
Assessment of adipogenic differentiation by Oil Red staining of lipid droplets. **(A,B)** Representative high-power fields of Oil red staining of HPS/PRP/NS-10%, −40% stimulated preadipocytes compared to negative control (growth media) at day 2 **(A)** and day 4 **(B)**. Scale bar = 50 μm. **(C)** Quantification of Oil red staining by optical density (OD). Two-way repeated-measures ANOVA with Tukey’s multiple comparisons test. Data points are means ± SEM, Preadipocyte donors: n = 3. Capital letter pairs over plots indicate statistical comparison of corresponding data points. For all pair comparisons, * = p < 0.05, # = p < 0.01, + = p < 0.001,: = p < 0.0001.

Adipogenic differentiation was assessed by Oil Red O staining to quantify intracellular lipid droplet accumulation (Kraus et al., 2016). A significant increase in lipid droplet formation was observed in all blood-derived conditions from day 2 to day 4 (Figure 4). Notably, preadipocytes stimulated with HPS-40% showed significantly higher Oil Red O staining already at day 2 compared to the other groups. This effect was further intensified by day 4, resulting in the highest overall lipid accumulation, with up to a 1.8-fold increase (Figures 4B,C).

### 3.4 Analysis of adipogenic gene expression

**FIGURE 5:**
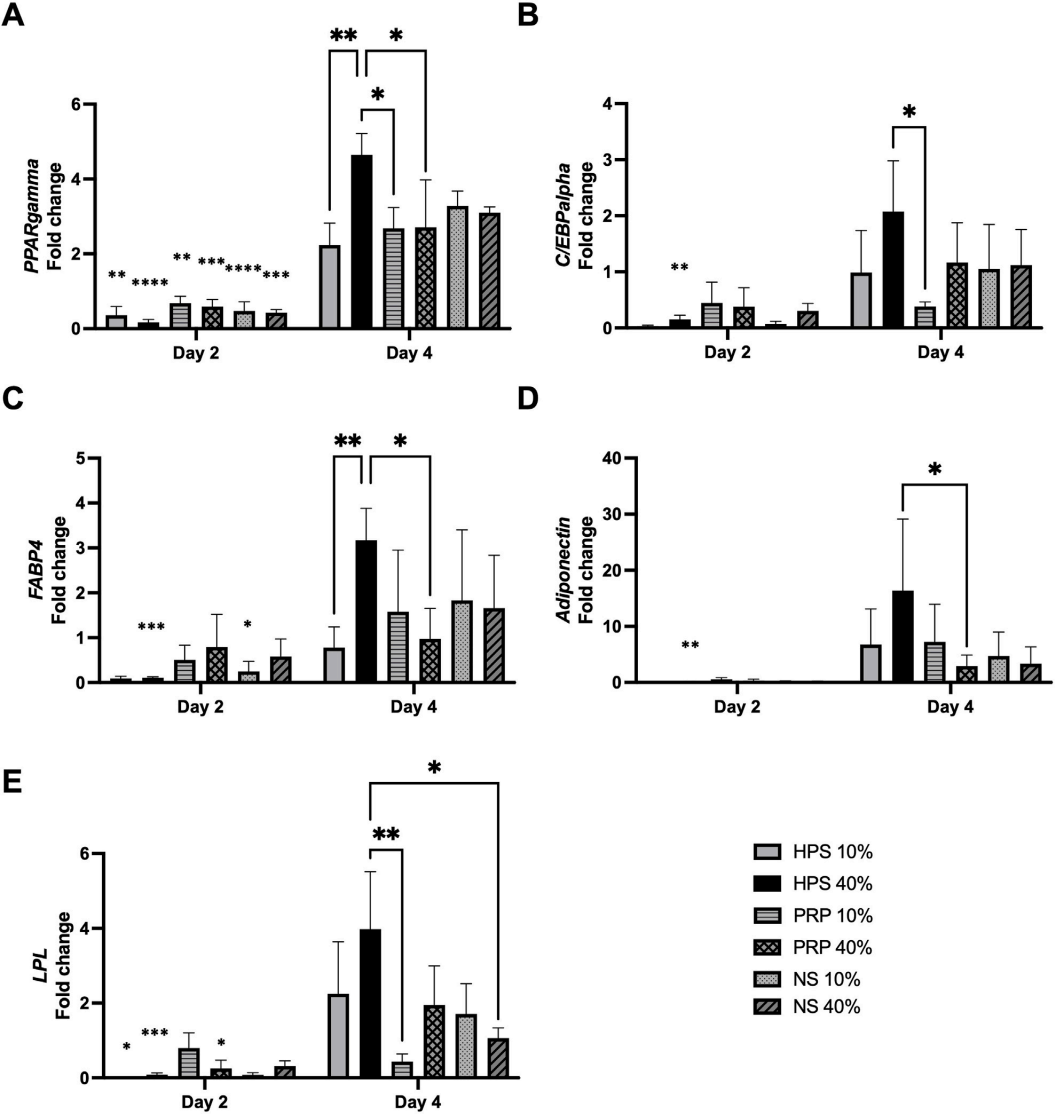
Gene expression analysis was performed using qRT-PCR and the resulted expression data were demonstrated as fold change of the control group (basal media). Gene expression of adipogenic-specific marker genes **(A)** PPARgamma, **(B)** C/EBPalpha, **(C)** FABP4, **(D)** Adiponectin, **(E)** LPL, at day 2 and day 4 of HPS/PRP/NS-10% and −40% stimulated preadipocytes. Two-way repeated-measures ANOVA with Tukey’s multiple comparisons test. Data points are means ± SEM, Preadipocyte donors: n = 3. *p < 0.05, **p < 0.01, ***p < 0.001, ****p < 0.0001. Asterisks without brackets indicate the pairwise comparison between the same condition of day 2 and day 4.

Finally, we analyzed the adipogenic gene expression of PPARgamma, C/EBPalpha, FABP4, Adiponectin, and LPL of the preadipocyte cultures. Here, we observed a significant increase of the adipogenic differentiation marker PPARgamma in all blood-derived conditions from day 2 to day 4, with HPS-40% treated preadipocytes reaching the highest expression (Figure 5A). For the other adipogenic markers (CEBP-alpha, FABP4, Adiponectin, and LPL), HPS-40% was also significant in increasing expression from day 2 to day 4 HPS-40% while it exhibited the most elevated expression of CEBP-alpha, FABP4, Adiponectin, and LPL on day 4 (Figures 5B–E). However, significance to the other conditions was not always observed.

## 4 Discussion

Supplementation with blood-derived growth factor-rich compositions has been proposed as an adjunctive strategy to enhance the survival and integration of autologous fat grafts. In this study, we evaluated the biological effects of Hypoxia Preconditioned Serum (HPS) and Platelet-rich Plasma (PRP) on human preadipocyte proliferation, viability, and differentiation *in vitro*. The results demonstrate HPS as an effective promoter of preadipocyte survival and adipogenic differentiation. Among the tested secretomes, HPS exhibited superior performance in enhancing lipid droplet formation and inducing robust upregulation of adipogenic markers. These findings support the potential of HPS as a promising therapeutic tool in regenerative medicine, particularly in optimizing the outcomes of adipose tissue grafting procedures.

Proteomic analysis revealed that HPS contains significantly higher concentrations of key adipogenic growth factors, including adiponectin, IGF-1, VEGF-A, bFGF, and PDGF-BB, compared to NS and PRP. These biomolecules play crucial roles in adipocyte lineage commitment, clonal expansion, and cellular survival (Jin et al., 2018; Song et al., 2014; Zhao et al., 2013; Benvie et al., 2024; Staiger and Löffler, 1998). An elevated level of these factors in HPS indicates an enriched adipogenic profile, suggesting that this secretome may offer superior regenerative capacity. Likewise, the dual angiogenic and adipogenic properties of VEGF-A, bFGF, IGF-1, and PDGF-BB are possibly advantageous *in vivo*, where vascularization is a limiting determinant of graft viability (Omorphos et al., 2021; Lin et al., 2017). This is particularly relevant in the context of the three-layered fat graft zone model (survival, regenerating, and necrotizing zone) described by Eto et al. (2012), wherein the regenerating zone is prone to oxygen and nutrient deprivation. Therapeutic angiogenesis may improve graft survival by supporting this vulnerable zone, though the full extent of its combined adipogenic and angiogenic effects can only be assessed *in vivo*. Interestingly, leptin concentrations were reduced in both HPS and PRP compared to NS. Although leptin is typically associated with energy homeostasis, it also plays a role in inflammation (Iikuni et al., 2008). In the setting of hypoxia or injury, leptin can exacerbate inflammatory cascades. Thus, its downregulation in HPS and PRP may favor tissue repair by mitigating the inflammatory burden, as suggested by other studies (Kiernan and MacIver, 2021).

Cell viability and proliferation assays further demonstrated that all secretomes (HPS, NS, and PRP) enhanced preadipocyte expansion without inducing cytotoxic effects. The highest proliferation rate was observed in the NS-40% group, while the HPS-40% group yielded slightly lower, but non-significant, cell counts, comparable to those in the preadipocyte growth medium (positive control). These results suggest that while HPS may not significantly increase cell proliferation compared to NS and PRP, it does not exert any detrimental effects on cell viability. It is worth noting that the increased proliferation induced by NS and PRP may reflect the expansion of undifferentiated progenitors rather than terminally differentiated adipocytes, which do not undergo mitosis (Lai et al., 2018; Middleton et al., 2012). This distinction is of the essence, as excessive proliferation without concurrent differentiation may not yield a stable graft volume. According to several studies, PRP inhibits adipogenic differentiation but promotes ASC and preadipocyte proliferation (Chignon-Sicard et al., 2017; Liao et al., 2015; Fukaya et al., 2012). This is possibly due to its platelet-derived growth factor dominance and lack of hypoxia-related signalling intermediates. Nevertheless, despite other reports of improved graft retention with PRP, its inconsistent influence on adipocyte lineage specification has made its role in lipofilling protocols contentious (Chignon-Sicard et al., 2017; Atashi et al., 2019; D'Esposito et al., 2015).

Further mechanistic insight was provided using Oil Red O staining and quantification. Results of HPS-40% treatment indicated a marked increase in lipid droplet formation at both 48 and 96 h, indicating its superior capacity to enhance adipogenic differentiation in preadipocytes in comparison to NS and PRP. Lipid accumulation is a functional hallmark of terminal adipocyte differentiation (Kraus et al., 2016), and its enhancement by HPS implies maturation toward a physiologically competent adipocyte phenotype. This may be attributed in part to elevated levels of growth factors such as IGF-1 and adiponectin, both of which are pivotal regulators of adipogenesis (Zhao et al., 2013; Fu et al., 2005). IGF-1, promotes the differentiation of preadipocytes into mature adipocytes by activating signaling pathways such as the PI3K/Akt pathway, which is essential for adipogenesis (Zhao et al., 2013). While adiponectin, on the other hand, promotes lipid metabolism in adipocytes, further contributing to the differentiation process (Fu et al., 2005). By contrast, the other groups did not show a marked difference in lipid accumulation compared to the negative control (growth media), suggesting that their protein factors had no substantial effect on preadipocyte differentiation. In line with the earlier observation of increased proliferation in these groups, this may indicate a predominance of proliferating preadipocytes rather than the existence of fully differentiated adipocytes. Compared to reports in the literature, PRP did not promote adipogenic differentiation in ASCs (D'Esposito et al., 2015; Cervelli et al., 2012) and instead appeared to induce a phenotypic shift toward myofibroblast-like cells (Chignon-Sicard et al., 2017).

The gene expression analysis provided additional evidence of the adipogenic effects of HPS, NS, and PRP. All conditions showed an increase in the expression of adipogenic markers such as PPARgamma, C/EBPalpha, FABP4, Adiponectin, and LPL from day 2 to day 4. These markers are well-established indicators of adipocyte differentiation and lipid metabolism (Kiernan and MacIver, 2021; Fu et al., 2005; Enerbäck et al., 1992; Furuhashi et al., 2014; Lee et al., 2016; Rosen et al., 2002). Markedly, preadipocytes treated with HPS-40% consistently exhibited the highest expression levels across most markers by day 4, reinforcing the conclusion that HPS facilitates transcriptional activation of the adipogenic program. Although not all comparisons were deemed statistically significant, the trend was biologically consistent with observed lipid accumulation. This favorable effect of HPS-40% has also been demonstrated in previous studies investigating its role in osteogenesis (Jiang et al., 2022a; Jiang et al., 2024b) and dermal regeneration (Jiang et al, 2022a; Jiang et al., 2024a). In contrast to a previous study reporting that treatment with at PRP-5% did not increase PPARgamma expression in ASCs compared to FCS-10% control media up to 6 days (Cervelli et al., 2012), our results indicated that on day 4, treatment with PRP-10% and −40% led to a 2.6-fold and 2.7-fold respective increase in PPARgamma expression in preadipocytes in comparison to FCS-3% control media. Apparently, more committed progenitor cells in the adipocyte lineage, i.e., preadipocytes, are more responsive in their differentiation potential. Indeed, it was shown, that PPARgamma-expressing preadipocytes are more adipogenic *in vitro* than PPARgamma-negative cells, while ASCs can differentiate into adipocytes but require additional signals for commitment (Cawthorn et al., 2012; Tang et al., 2008). These findings draw attention to the importance of selecting appropriately committed cell populations for evaluating adipogenic stimuli. Further research is needed to investigate the signaling pathways activated by HPS in preadipocytes and to elucidate the mechanisms by which these pathways contribute to the observed effects on adipogenesis.

Taken together, the findings of this study suggest that HPS exerts a favourable effect on adipogenic differentiation of preadipocytes while maintaining cell viability *in vitro*. Its enriched composition of hypoxia-inducible growth factors may help address the metabolic and vascular challenges inherent to lipofilling procedures. From a translational perspective, HPS therefore represents a promising candidate for further investigation as a bioactive supplement to fat grafting. Given its angiogenic potential (Jiang et al. 2022b; Moog et al., 2023), its use could also improve revascularisation of grafted tissue *in vivo*, facilitating long-term survival and volume maintenance. So far, the current data are limited to a monoculture model of committed preadipocytes and cannot directly predict outcomes in the complex *in vivo* environment of adipose grafts. Future studies should focus on validating these findings in animal models and clinical trials to determine the efficacy, mechanisms, and volumetric requirement (%) of HPS in promoting adipose tissue engraftment. To date, only PRP has been comprehensively investigated both preclinically and clinically. A review of 11 studies involving 1,125 patients found that combining PRP with fat grafting significantly improved fat graft survival rates (up to 89.2%) and reduced recovery time compared to fat grafting alone (Wu et al., 2021). Histology analysis of the transplanted fat with PRP-20% in mice revealed enhanced neovascularization beginning at 4 weeks and persisting through 12 weeks post-transplantation (Xiong et al., 2018). While another study demonstrated that administering PRP twice, on the day of transplantation and again on day 14, significantly enhanced long-term fat graft retention and angiogenesis compared to a single PRP injection or fat grafting alone (Li et al., 2020). This highlights the importance of revascularization as an additional key factor in graft survival, potentially outweighing the role of direct adipocyte survival. Given that PRP-enhanced grafting appears to be more effective *in vivo* than *in vitro*, a direct comparison with HPS-supplemented lipofilling is warranted to better understand the relative benefits of each approach. Elucidating their relative efficacy could establish a new benchmark for biological enhancement of fat grafting and offer refined strategies for regenerative soft tissue reconstruction.

It must be mentioned that the present study did not assess *de novo* adipogenic induction. Demonstrating such lineage commitment would require the use of ASCs, which are capable of differentiating into multiple cell types. However, our rationale was translational: The experiments were designed to assess how HPS and PRP influence proliferation, survival, and terminal adipogenic differentiation of cells already committed to the adipocyte lineage. Another limitation is the absence of a standard adipogenic differentiation cocktail as a positive control. While such pharmacological cocktails (e.g., IBMX, dexamethasone, insulin, or PPARgamma agonists) are commonly used to achieve maximal induction *in vitro*, our aim here was to examine the translational relevance of HPS and PRP under conditions closer to their potential clinical application.

## Data Availability

The raw data supporting the conclusions of this article will be made available by the authors, without undue reservation.
